# Brain Connectomics Improve the Prediction of High-Risk Depression Profiles in the First Year following Breast Cancer Diagnosis

**DOI:** 10.1155/2024/3103115

**Published:** 2024-05-17

**Authors:** Mu Zi Liang, Peng Chen, Ying Tang, Xiao Na Tang, Alex Molassiotis, M. Tish Knobf, Mei Ling Liu, Guang Yun Hu, Zhe Sun, Yuan Liang Yu, Zeng Jie Ye

**Affiliations:** ^1^Guangdong Academy of Population Development, Guangzhou, China; ^2^Basic Medical School, Guizhou University of Traditional Chinese Medicine, Guiyang, China; ^3^Institute of Tumor, Guangzhou University of Chinese Medicine, Guangzhou, China; ^4^Shenzhen Bao'an Traditional Chinese Medicine Hospital, Guangzhou University of Chinese Medicine, Shenzhen, China; ^5^College of Arts, Humanities and Education, University of Derby, Derby, UK; ^6^School of Nursing, Yale University, Orange, CT, USA; ^7^Sun Yat-sen University Cancer Center, State Key Laboratory of Oncology in South China, Collaborative Innovation Center for Cancer Medicine, Guangzhou, China; ^8^Army Medical University, Chongqing Municipality, China; ^9^The First Affiliated Hospital, Guangzhou University of Chinese Medicine, Guangzhou, China; ^10^South China University of Technology, Guangzhou, China; ^11^School of Nursing, Guangzhou Medical University, Guangzhou, Guangdong Province, China

## Abstract

**Background:**

Prediction of high-risk depression trajectories in the first year following breast cancer diagnosis with fMRI-related brain connectomics is unclear.

**Methods:**

The Be Resilient to Breast Cancer (BRBC) study is a multicenter trial in which 189/232 participants (81.5%) completed baseline resting-state functional magnetic resonance imaging (rs-fMRI) and four sequential assessments of depression (T0-T3). The latent growth mixture model (LGMM) was utilized to differentiate depression profiles (high vs. low risk) and was followed by multivoxel pattern analysis (MVPA) to recognize distinct brain connectivity patterns. The incremental value of brain connectomics in the prediction model was also estimated.

**Results:**

Four depression profiles were recognized and classified into high-risk (delayed and chronic, 14.8% and 12.7%) and low-risk (resilient and recovery, 50.3% and 22.2%). Frontal medial cortex and frontal pole were identified as two important brain areas against the high-risk profile outcome. The prediction model achieved 16.82-76.21% in NRI and 12.63-50.74% in IDI when brain connectomics were included.

**Conclusion:**

Brain connectomics can optimize the prediction against high-risk depression profiles in the first year since breast cancer diagnoses.

## 1. Introduction

Breast cancer accounts for 24.5% of all new female cancer cases worldwide, with an increasing incidence trend in China [1]. Improved screening and innovative treatments have resulted in a 5-year survival rate of >90% in Westernized countries and increasing to >80% in China [2, 3]. There are known persistent physical and psychological symptoms among breast cancer survivors. Compared with healthy women, a higher prevalence of depression (10-25%) was identified in survivors of breast cancer within the first year since diagnosis [4, 5]. Depressive symptoms are associated with poor treatment adherence, increased suicide ideation, and lower quality of life [6, 7]. Early recognition of cancer survivors with depressive symptoms is needed, especially research to identify severity and patterns across time [8, 9].

However, predicting depression profiles that may be at higher risk remains difficult in the context of a complex of factors, such as demographics, psychosocial distress, social support, and clinical characteristics [10–12]. More objective approaches have been studied, specifically using brain imaging. Reduced amygdala and hippocampus volume were reported to be associated with depression after breast cancer, but the causal association could not be concluded due to cross-sectional study designs [13, 14]. The wide application of noninvasive resting-state functional magnetic resonance imaging (rs-fMRI) offers an opportunity to evaluate brain connectomics using data-driven multivoxel pattern analysis (MVPA) which is designed to reduce false positives or negatives in fMRI-related analysis [15–17]. To our knowledge, no previous research has been conducted to estimate the associations between brain connectomics and high-risk depression profiles in breast cancer.

We hypothesized that (1) distinct depression profiles (high and low risk) would be identified in the first year of breast cancer, (2) brain connectomics would be a robust predictor of a high-risk depression profile, and (3) brain connectomics could provide incremental predicting power over the conventional TNM staging system.

## 2. Method

### 2.1. Sample

**Figure 1:**
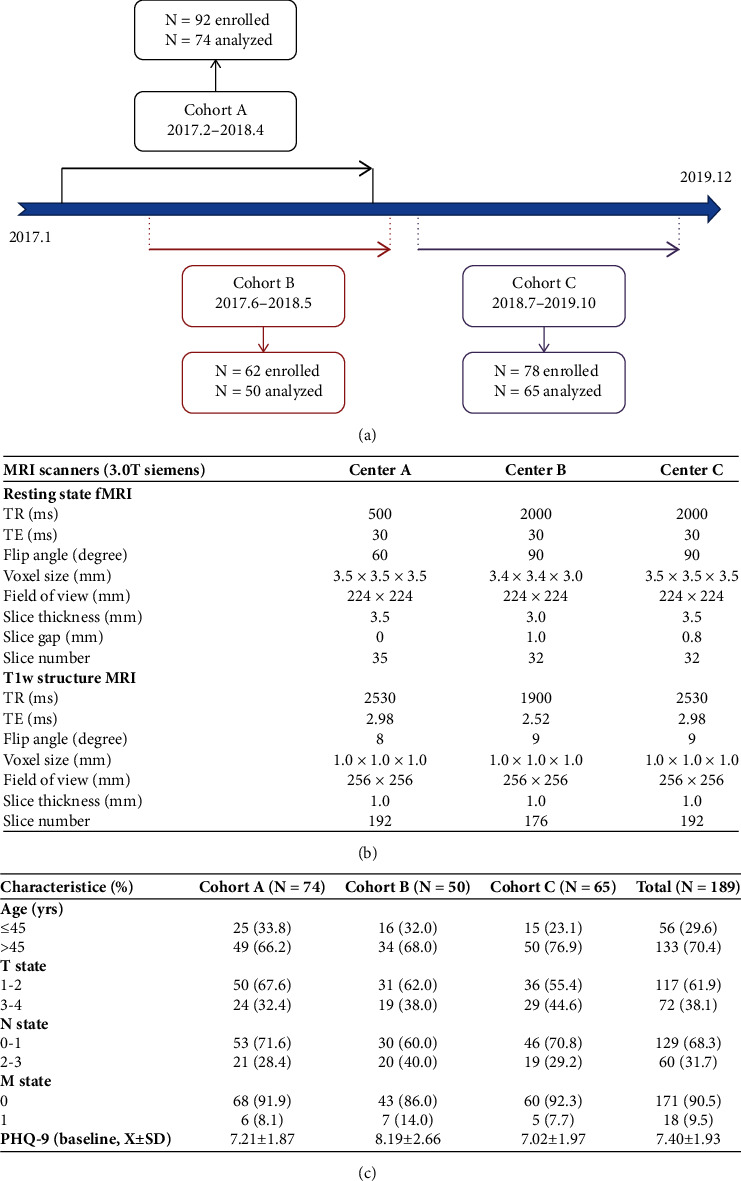
(a) Enrollment line in the Be Resilient to Breast Cancer (BRBC). (b) fMRI parameters in different centers. (c) Demographic and clinical characteristics of participants.

The Be Resilient to Breast Cancer (BRBC) is a multicenter longitudinal study, and 189/232 participants (81.5%) completed depression questionnaires and fMRI imaging (Figure 1(a)). Three centers (cohorts A, B, and C) from different cities (Guangzhou, Shenzhen, and Foshan) were involved, and details have been described elsewhere [18–22]. The inclusion criteria for patients were as follows: (1) aged > 18 years, (2) fluent in Mandarin or Cantonese, and (3) informed consent. The exclusion criteria were as follows: (1) life expectancy less than 12 months and (2) declined to participate in the current study. fMRI data were obtained at baseline (T0), and research nurses were trained to collect depression data at different assessments (T0-T3). To achieve a high response rate, both offline and online evaluations were available for data collection on depression. There was ethics approval for the study (2016KYTD08).

### 2.2. Data Collection

#### 2.2.1. Patient Health Questionnaire-9 (PHQ-9)

The PHQ-9 is a 9-item scale about depressive symptoms based on the DSM-IV criteria [23]. The total score ranges from 0 to 27, with a higher score indicating a higher level of depressive symptoms. A cut-off of 10 indicates a potential major depressive disorder [24]. In the current study, Cronbach's coefficient was 0.84.

#### 2.2.2. MRI Data Acquisition and Preprocessing

3.0T Siemens were used as MRT scanners across different centers, and the parameters are detailed in Figure 1(b). SPM12 was utilized in the current study to handle slice timing correction, smoothing, and other spatial preprocessing procedures. In addition, BOLD timeseries within CSF and white matter and artifactual covariates were regressed out as temporal covariates. The band-pass filter was set at 0.01 Hz < *f* < 0.10 Hz.

### 2.3. Data Analysis

#### 2.3.1. Latent Growth Mixture Model (LGMM) for Depression Trajectories

**Figure 2:**
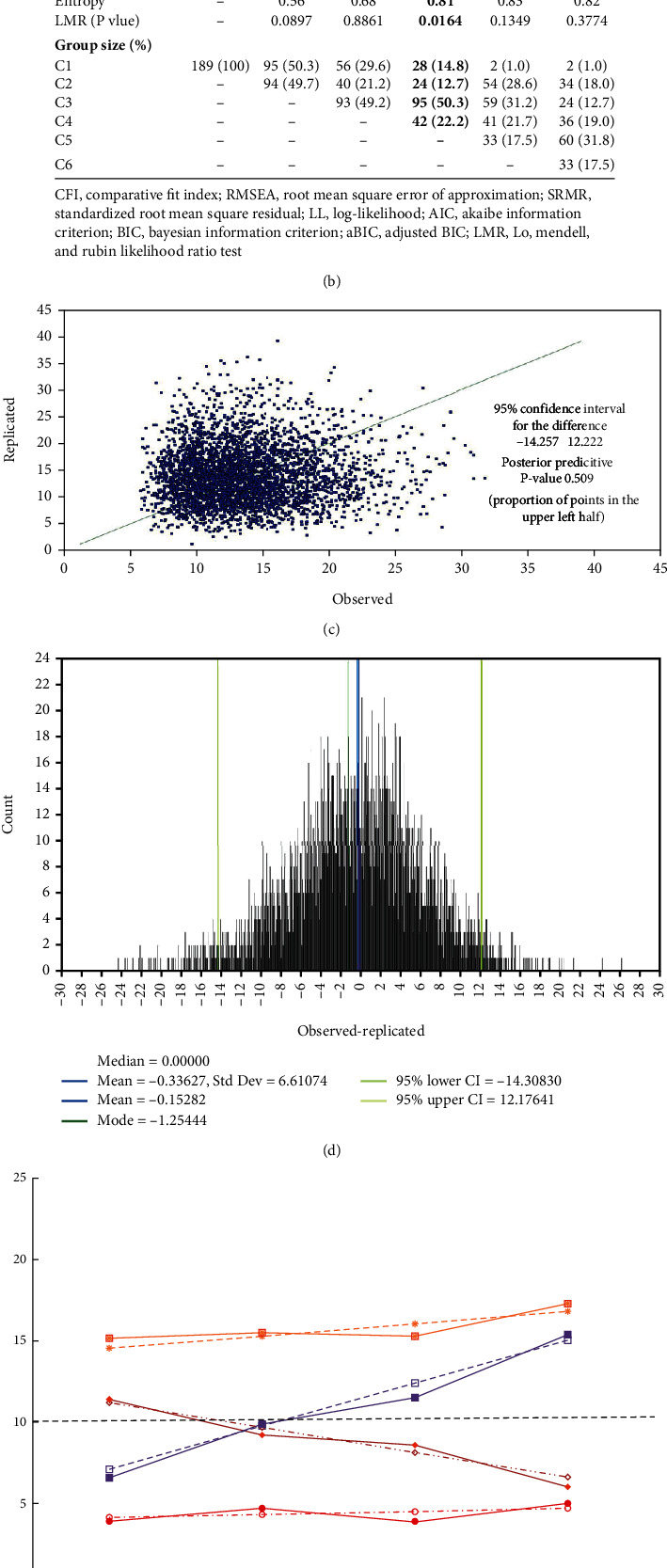
(a) Hypothesized model for LGMM. (b) Model fittings for unconditional LGMM. (c) Bayesian posterior predictive checking scatterplots. (d) Bayesian posterior predictive checking distribution. (e) Trajectory patterns for four distinct depression trajectories.

First, linear and nonlinear LGMM were compared in consideration of fitting indicators of comparative fit index (CFI), root mean square error of approximation (RMSEA), standardized root mean square residual (SRMR), Bayesian information criterion (BIC), and model simplification [25]. Three parameters of intercept (I), linear growth (Slope_S), and nonlinear growth (Slope_Q) were estimated based on the depression data at different assessments (PHQ_1, PHQ_2, PHQ_3, and PHQ_4). In addition, measurement errors were also considered (Error_1, Error_2, Error_3, and Error_4). Second, when the optimal LGMM was determined, another parameter of subgroups (class) was taken into consideration to explore the heterogeneity in the baseline (intercept) and growth (slope). The class number was increased from 1 to 6 according to the Akaike information criterion (AIC), BIC, adjusted BIC (aBIC), entropy value, and Lo-Mendell-Rubin likelihood ratio test (LMR) [26]. Maximum likelihood estimation was performed, and the results were further validated by a Bayesian analysis of four independent Markov chain Monte Carlo (MCMC) chains. A hypothesized LGMM for depression trajectories with four assessments is detailed in Figure 2(a).

#### 2.3.2. Data Analysis Process

First, LGMM was performed for PHQ-9 data with four assessments. In the current study, four distinct depression trajectories (resilient, recovery, delayed, and chronic) were recognized and classified into high-risk (resilient and recovery, coded as 1) and low-risk (delayed and chronic, coded as 0) trajectories. Second, a multivoxel pattern analysis (MVPA) was performed by using baseline fMRI data to predict the trajectory outcome (high vs. low risk) [27]. In consideration of small sample sizes in different centers, a conservative ratio of 10 : 1, 20 : 1, 30 : 1, and 40 : 1 was explored, resulting in a retained component number of 7, 4, 3, and 2, respectively (MVPA_7, MVPA_4, MVPA_3, and MVPA_2) [28]. In addition, the prediction abilities of different MVPA-based models including MVPA_2 vs. MVPA_3, MVPA_2 vs. MVPA_4, and MVPA_2 vs. MVPA_7 were further compared in order to get the optimal component, and NRI (net reclassification improvement) as well as IDI (integrated discrimination improvement) were calculated [29, 30]. Third, when the optimal MVPA component was determined, taking cohort A as the training dataset, brain connectivity was estimated and a mask template was extracted from those significant brain areas (height threshold uncorrected *p* < 0.01; cluster-level FDR-corrected *p* < 0.05), which was further utilized as the region of interest (ROI) in cohorts B and C as the validation dataset. Then, a new model combining TNM stage and brain values from ROI (Model 2) was compared with the conventional TNM stage model (Model 1) in consideration of AUC, NRI, IDI, calibration and decision curves, and clinical impact curves according to TRIPOD guideline [31–33]. Mplus was used for LGMM analysis, while MATLAB R2021b, SPM 12, and CONN software were utilized for fMRI-related analysis. R was used for the development and validation of prediction models.

## 3. Results

### 3.1. Demographic and Clinical Characteristics

In Figure 1(a), 74/92 (80.4%), 50/62 (80.6%), and 65/78 (83.3%) participants had complete depression and fMRI data. There were no significant differences in demographics or clinical characteristics between those with incomplete versus complete data (all *p* > 0.05). Other information is described in Figure 1(c).

### 3.2. LGMM for Depression Profiles

Compared with a nonlinear model, the linear one was chosen in consideration of the relative fitting indicators of CFI, RMSEA, SRMR, BIC, and model simplification (Figure 2(a)). In Figure 2(b), the class number was increased from 1 to 6, and four distinct depression trajectories including resilient (*N* = 95, 50.3%), recovery (*N* = 42, 22.2%), delayed (*N* = 28, 14.8%), and chronic (*N* = 24, 12.7%) were recognized as the optimal LGMM according to AIC, BIC, aBIC, and LMR (*p* value). The entropy value of 0.81 indicated a high accuracy (>90%) and was further validated by a Bayesian analysis of four independent MCMC chains (Figures 2(c) and 2(d)). In Figure 2(e), intercept and slope (variances) parameters for four distinct depression trajectories were described.

At last, the four trajectories were classified into high (resilient and recovery, coded as 1) and low (delayed and chronic, coded as 0) risk profiles.

### 3.3. Multivoxel Pattern Analysis (MVPA) and Significant Brain Areas

**Figure 3:**
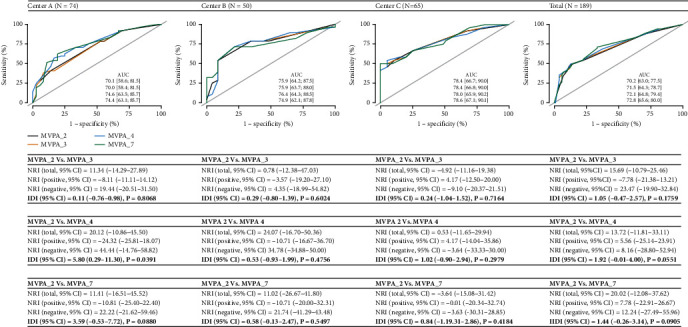
The comparison of different models in multivoxel pattern analysis (MVPA) across different centers.

**Figure 4:**
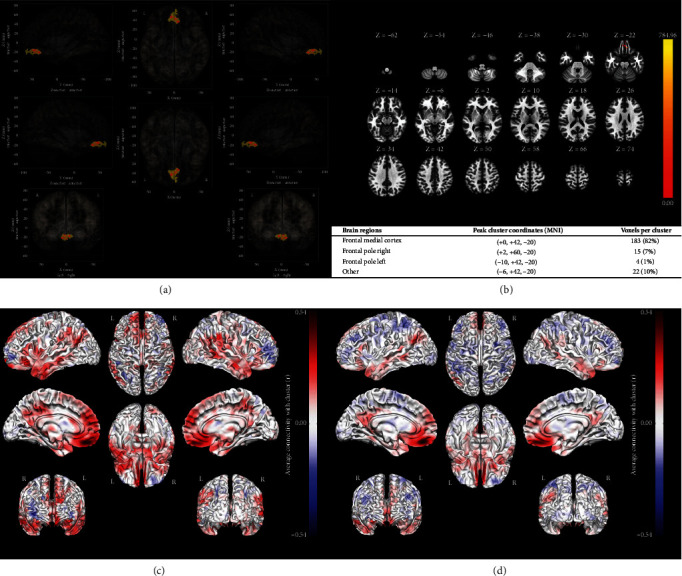
(a) Significant brain regions in MVPA_2 (frontal medial cortex and frontal pole). (b) Peak Cluster Coordinates (MNI) and voxels per cluster for significant brain regions. (c) Seed-to-voxel associations in the high-risk depression trajectory. (d) Seed-to-voxel associations in the low-risk depression trajectory.

In Figure 3, different component numbers were explored in MVPA against the trajectory outcome across different centers. A two-component model (MVPA_2) was chosen as the optimal MVPA in consideration of the nonsignificant increase in NRI (ranged from -4.92% to 24.07%) and IDI (ranged from 0.11% to 5.80%) when the component number was increased from 2 to 7 as well as a low false positivity. Two brain regions including (1) frontal medial cortex and (2) frontal pole left and right were identified as significant areas, which are presented in Figure 4(a). Peak Cluster Coordinates (MNI) and voxels per cluster are described in Figure 4(b), and the difference of brain connectivity between high- and low-risk trajectories is visualized in Figures 4(c) and 4(d) using ROI derived from MVPA.

### 3.4. The Prediction Model Combining TNM Stage and Brain Connectomics

**Figure 5:**
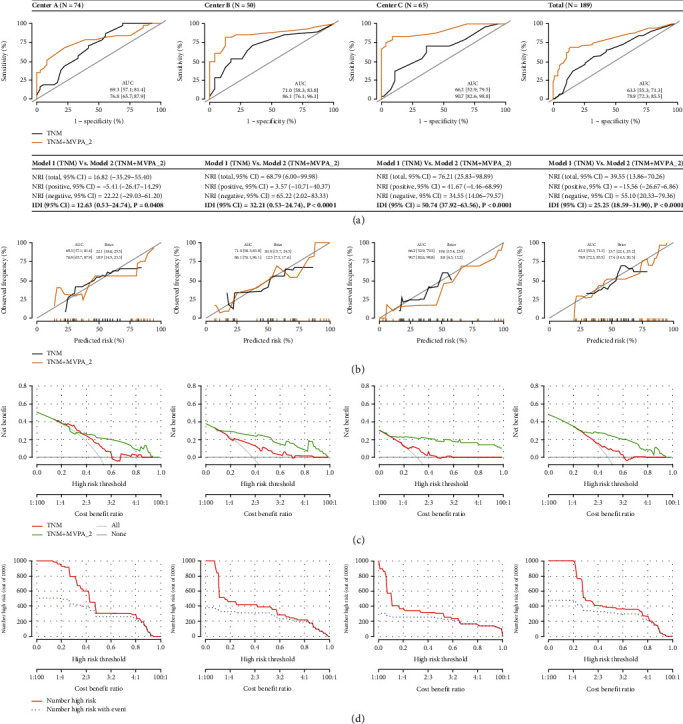
(a) AUC, NRI, and IDI for Model 1 (TNM stage) and Model 2 (TNM stage + brain connectomics). (b) Calibration curves for Model 1 and Model 2. (c) Decision curve analysis for Model 1 and Model 2. (d) Clinical impact curve for Model 2.

Compared with Model 1 (TNM stage), AUC in Model 2 (TNM stage + brain connectomics) increased from 63.3-71.0% to 76.8-90.7% when the brain values were included in the regressions (Figure 5(a)). In addition, NRI and IDI ranged from 16.82 to 76.21% and 12.63 to 50.74%, respectively (Figure 5(a)). In Figure 5(b), compared with 19.6-23.7 in Model 1, less Brier scores of 8.8-18.9 were recognized in Model 2, indicating a better fit. In Figure 5(c), compared with Model 1, higher net benefits were also identified in Model 2 across different risk thresholds. In Figure 5(d), clinical impact curves were visualized for Model 2 to facilitate its clinical utilization across different risk thresholds.

## 4. Discussion

First, the Be Resilient to Breast Cancer (BRBC) cohort was used to explore the associations between brain connectomics and high-risk depression profiles of women diagnosed with breast cancer within the first year. Four distinct depression profiles were identified, which were consistent with previous research [34]. Approximately 25% of participants in our study were classified into high-risk depression profiles (delayed or chronic). It is important to note the delayed profile and benefit of the longitudinal data collection and analysis due to the presence of low depressive symptoms at baseline.

Second, different from most previous research, participants enrolled in the Be Resilient to Breast Cancer (BRBC) study did not receive chemotherapy and would not be affected by common neurotoxicity [35]. Our results showed significant differences in the frontal medial cortex and frontal pole between patients with high- and low-risk depression profiles. Frontal areas are associated with cognitive function, and breast cancer survivors were reported to need more effort and time to complete cognitive tasks which provided evidence for the depression trajectories [36]. Without an a priori theoretical hypothesis, MVPA was performed in the current study to achieve a maximized prediction ability of high-risk depression profiles. However, significant brain areas identified by MVPA could not always be well explained. For instance, in Figure 4(b), 22 voxels (10%) could not be anatomically labelled in MNI, although these voxels survived correction for multiple comparisons (height threshold uncorrected *p* < 0.01; cluster-level FDR-corrected *p* < 0.05). Therefore, the difference should be further compared between an a priori anatomical approach (i.e., ROI-to-ROI/seed-based connectivity analyses) and an agnostic MVPA approach in future research.

Third, the results showed that rs-fMRI neuromarkers were robust predictors for a high-risk depression profile and could contribute incremental predicting power over conventional TNM staging systems. When rs-fMRI neuromarkers and TNM staging were combined, the patients were successfully classified into high- and low-risk depression profiles with approximately 76.8-90.7% accuracy. Thus, in consideration of easy access across different medical centers and the noninvasive nature of data collection, rs-fMRI could be considered in the precision management approach of breast cancer [37, 38]. Furthermore, the reliability of rs-fMRI neuromarkers was quite high across different datasets in the present study, and the prediction ability of baseline neuromarkers has previously been validated in populations with depression, generalized anxiety, and schizophrenia [39–42]. In addition, using the anisotropic Brownian motion of water molecules along the nerve fiber method, diffusion tensor imaging (DTI) could be considered in the prediction model as a noninvasive tractography indicator and could provide unique information about brain structure in future research [43]. However, due to the complex nature of the brain, opposite results are often recognized between fMRI-related connectivity and DTI-related ones, even within the same samples [44, 45]. Thus, whether multimodal brain connectomics could achieve a better prediction ability should be further investigated.

## 5. Limitations

Several issues should be considered in the current study. First, patients were enrolled in one of the most developed provinces in China, and the LGMM-related findings might not be generalizable to patients across varied socioeconomic statuses. Second, previous psychiatric diagnosis and hospitalization could not be determined from the medical chart which will affect the findings here [46]. Second, in consideration of the restriction of the unconditional model in LGMM, many confounders (i.e., antidepressant prescription, family history of psychiatry disorder) are not considered in MVPA, resulting in increased type I errors. A priori anatomical approach could be performed to validate MVPA-related findings. Third, the assumption of the LGMM may be compromised due to the time difference in measurements between subjects across the observation period, and modeling errors should be noted here. Fourth, the sample sizes of the training and validation cohorts are relatively small, and the prediction model should be validated in a larger population with different cancer diagnoses. Fifth, measurement errors were not taken into consideration to calculate the QoL outcomes, and item response theory could be tried to achieve a better estimation [47–50].

## 6. Conclusion

Brain connectomics has been shown to predict high-risk depression profiles in women diagnosed with breast cancer within the first year.

## Data Availability

The data that support the findings of this study are available on request from the corresponding author. The data are not publicly available due to privacy or ethical restrictions.
